# Marine cloud brightening: regional applications

**DOI:** 10.1098/rsta.2014.0053

**Published:** 2014-12-28

**Authors:** John Latham, Alan Gadian, Jim Fournier, Ben Parkes, Peter Wadhams, Jack Chen

**Affiliations:** 1 National Centre for Atmospheric Research Boulder, CO 80301, USA; 2 School of Earth and Atmospheric Sciences, University of Manchester, Manchester M13 9PL, UK; 3 NCAS, SEE, University of Leeds, Leeds, LS2 9JT, UK; 4 Planetwork San Francisco, CA 94102, USA; 5 LOCEAN, Universite du Pierre et Marie Curie, Paris 75252, France; 6 Ocean Physics, DAMTP, University of Cambridge, Cambridge CB3 0WB, UK

**Keywords:** Arctic, Antarctic, polar sea-ice, recovery, preservation

## Abstract

The general principle behind the marine cloud brightening (MCB) climate engineering technique is that seeding marine stratocumulus clouds with substantial concentrations of roughly monodisperse sub-micrometre-sized seawater particles might significantly enhance cloud albedo and longevity, thereby producing a cooling effect. This paper is concerned with preliminary studies of the possible beneficial application of MCB to three regional issues: (1) recovery of polar ice loss, (2) weakening of developing hurricanes and (3) elimination or reduction of coral bleaching. The primary focus is on Item 1. We focus discussion herein on advantages associated with engaging in limited-area seeding, regional effects rather than global; and the levels of seeding that may be required to address changing current and near-term conditions in the Arctic. We also mention the possibility that MCB might be capable of producing a localized cooling to help stabilize the West Antarctic Ice Sheet.

## 1. Introduction

Two classic cloud physics studies [1,2] provided the physical background for the formulation [3,4] of the marine cloud brightening (MCB) climate engineering technique. The idea behind MCB is to seed marine stratocumulus clouds with substantial concentrations of roughly monodisperse, sub-micrometre seawater particles of sufficient salt mass to be activated to form additional cloud droplets and therefore to enhance the cloud droplet concentration N, and thus cloud albedo. Provided that the salt masses are not too high, the seeding could also enhance cloud longevity, because the production of drizzle drops would be inhibited.

A significant number of papers on MCB (e.g. [5–13]) have been published. Most attention has been focused on whether MCB—subject to various caveats, and resolution of technological difficulties—might possibly produce a global cloud albedo change of significance. Opinion is divided on this point. More specific work concerns the influence of MCB on rainfall, sea-ice cover and other aspects of climate. Several general circulation model (GCM) computations indicate that the associated cooling might be sufficient to balance global warming up to at least the carbon dioxide-doubling point (CO_2_×2), while other work [14] indicates that the required value of N may not be achievable.

**Figure 1.**
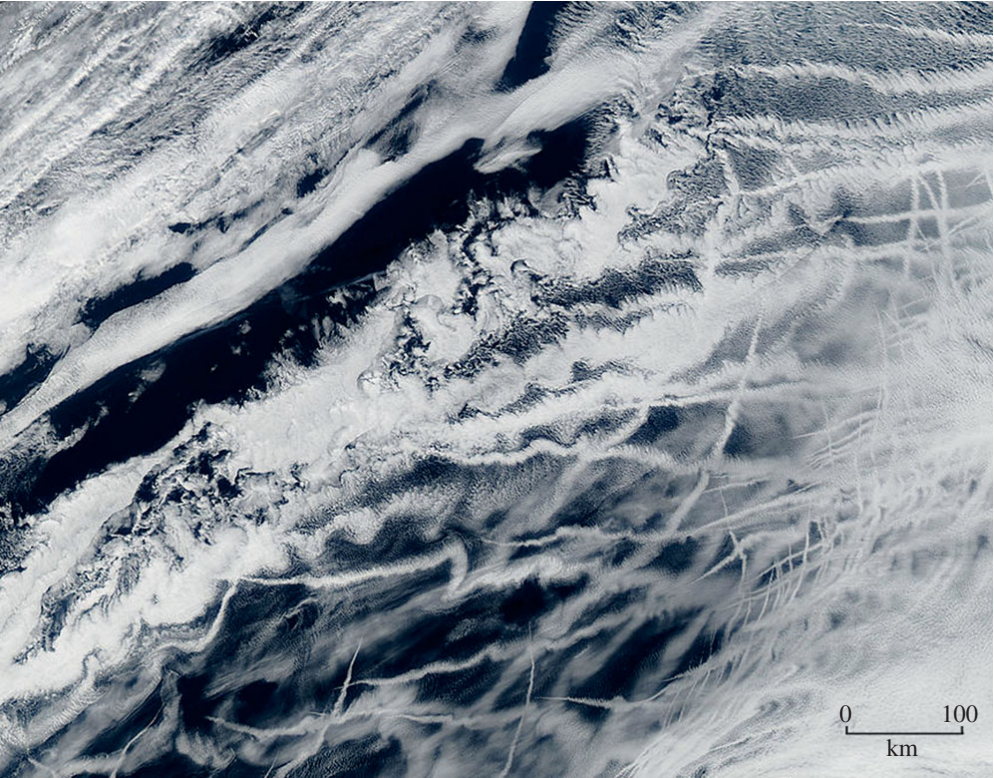
Ship-tracks over the Atlantic, 44°N–50°N by 5°W–15°W, west of the Bay of Biscay, France [15].

Ship-tracks (figure 1), which are commonly observed in satellite photographs, provide evidence supporting the contention that the albedo of maritime clouds can be appreciably enhanced by seeding. The production of ship-tracks indicates that significant albedo enhancement of some maritime clouds, can be produced, when seeded by sulfate emissions from ships. They do not form everywhere. It is unclear whether sea-salt seeding via MCB spraying could produce a similar degree of albedo enhancement, as field assessment of MCB will require spray technology capable of producing copious quantities of roughly monodisperse 100–800 μm diameter seawater spray micro-droplets. However, recent laboratory developments concerning spray-particle production are encouraging (A. Neukermans 2014, personal communication).

## 2. Possible regional applications of marine cloud brightening

Solar radiation management (SRM) climate engineering techniques have been more-or-less completely focused on global-scale issues. This has been the case with MCB since its inception. However, MCB differs from some other SRM strategies in that it could in principle also be deployed on a much less than global scale, thus offering a range of regional applications. On the one hand, it could possibly produce a localized ocean surface cooling, which could which could be applied to mitigate, or even avert, certain regional consequences of warming, which are being observed now, or which are predicted to occur in coming decades. On the other hand, MCB could be found to have deleterious consequences.

The regional application which constitutes the primary focus of this paper is the maintenance and restoration of Arctic and Antarctic sea ice. As part of that effort, we also present a new idea outlining how a focused application of MCB in warm southern oceans might help avert the collapse of the West Antarctic Ice Sheet. In addition to the above-mentioned primary work, we also present modest extensions of our published work on MCB-induced reduction of the strength of developing hurricanes and the preservation of coral reefs.

The studies outlined above have in common the MCB-induced reduction of sea surface temperatures (SSTs) in selected regions. The fact that all three above-mentioned applications of MCB involve reductions in SST does not indicate that other parameters do not play a significant role. They unquestionably do—but they lay beyond the scope of this preliminary study.

The seeding areas covered in this earlier work ranged considerably from 100% of all suitable maritime cloud regions [9–11] to the three areas containing the most extensive concentrations of such clouds—off the West coasts of North and South America, and Africa [7,8,11]. These three areas (combined) cover approximately 5% of the total oceanic area and were used in most of the current computations outlined herein. Marine stratocumulus clouds cover about 20% of the Earth's surface, with the oceans covering 71% of the globe. The cloud location is variable.

The climate model employed in §2*a*–*c* is the Meteorological Office Unified Model 6.1 (HadGEM1) [16]. It is a fully coupled atmosphere–ocean model with an interactive sea-ice parametrization. The atmosphere has 38 vertical levels up to 39 km and a horizontal grid of 1.25° of latitude by 1.875° of longitude. The ocean has 40 levels to a maximum depth of 5 km, and the horizontal resolution of the ocean is 1° by 1° between 30° and the poles, with resolutions gradually increasing to 1/3° meridionally between 30° and the equator. MCB was simulated by forcing the cloud droplet number concentration, N, of all maritime stratocumulus clouds to be 375 per cc within the three regions mentioned earlier. A technique for producing this spray rate has not yet been achieved. It is likely that the value of N required could be significantly smaller in some circumstances.

The three simulations used in our current studies are (1) a control (CON) with carbon dioxide levels held at a 2020 projected level of 440 ppm; (2) a climate change (CC) simulation, where the carbon dioxide fraction increases by 1% p.a. until 2045, where it is held steady at 560 ppm for the duration of the simulation, until 2090; and (3), an MCB simulation, which is the same as the [CC] simulation except that it includes seeding in the three stratocumulus regions defined above. All simulations were run for 70 model years with the final 20 years (2070–2090) used for analysis.

The studies outlined in §2*a*–*c* used three patches seeding (MCB3) as defined above. The Antarctic portion of §2*a* used all-ocean (MCBA) seeding.

### (a) Polar sea-ice maintenance or restoration via marine cloud brightening

Sea-ice coverage, averaged over consecutive years, is shrinking, as a consequence of the warming caused by increasing atmospheric carbon dioxide concentrations [17,18]. This also causes a positive feedback process to occur, because the melting of the (often snow-covered) sea ice produces a decrease in surface reflectivity from roughly 90% to 10% [19]. This reduction produces significant warming, with concomitant enhanced melting, which has recently been quantified [20]. For the period 1979–2011, warming due to reduced albedo in the Arctic produced a forcing of 6.4±0.9 W m^−2^, equivalent to an increase in 0.21±0.03 W m^−2^ averaged over the globe.

**Figure 2.**
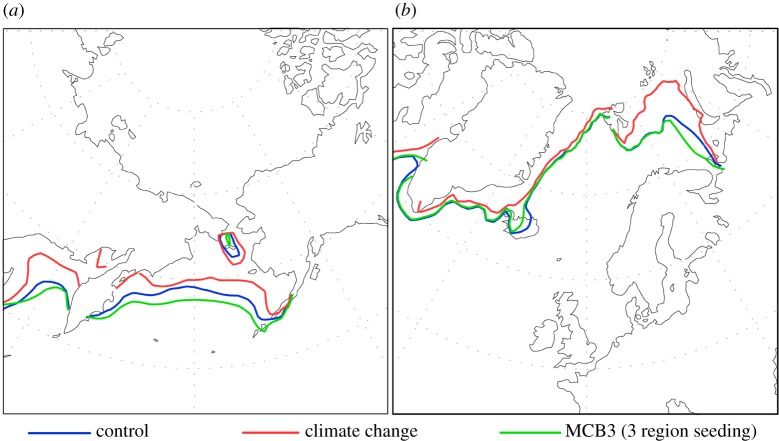
(*a*) Western Arctic (North Pacific) and (*b*) Eastern Arctic (North Atlantic) summer sea ice extent (JJA) between 2070 and 2090 for three climate model simulations: control (CO_2_=440 ppm); climate change (CO_2_= 560 ppm); MCB3 (three region seeding, CO_2_=560 ppm).

Our MCB simulations showing the extent of the Arctic Ice Fields in June July, August (JJA) are presented in figure 2. JJA is chosen to represent average seasonal changes, rather than the variable September minimum, which as such, may not be as representative of the slower climatic changes. In both the Northern and Southern Hemisphere winters, the CC scenario causes ice to retreat with respect to the CON. The MCB simulations recover much of the winter ice extent lost by the CC scenario. The majority of the winter ice loss in the CC simulation is in the North Pacific. By contrast, MCB extends the extent of ice cover further south. MCB in the summer months produces an ice-limit 50 km further south than the control in the Pacific, and close to the control in the Atlantic Ocean (figure 2).

The relevance of the above findings is dependent upon the accuracy of sea-ice change values generated in HadGEM1. The model does not generally produce significant changes in sea ice as a result of small perturbations in radiative forcing. In the Northern Hemisphere, the summer retreat in the CC simulation is 100 km greater than for MCB -CC, but only 50 km greater than for the control. In the Southern Hemisphere summer, the ice limit is not significantly different between the control, MCB and CC simulations (results not shown in this paper).

**Figure 3.**
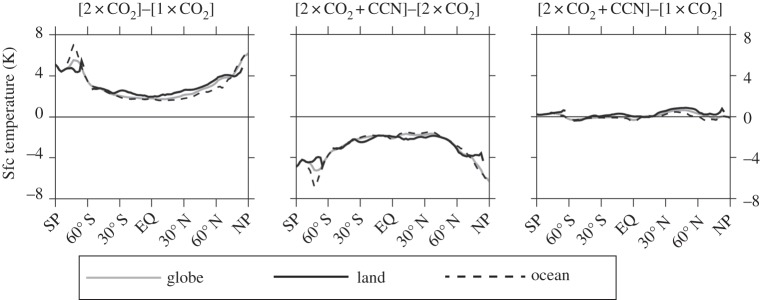
Changes in average annual mean surface temperature due to increase in CO_2_ (2×CO_2_−1×CO_2_), MCB (2×CO_2_+CCN−2×CO_2_), and 2×CO_2_ and MCB combined (2×CO_2_+CCN−1×CO_2_) for land (solid black), ocean (dashed) and all regions (grey).

A previous full-ocean MCB study (fig. 8, [12]), an excerpt from which is reproduced here in figure 3, shows that although increasing CO_2_ produces a maximum temperature rise at the poles, MCB yields an equivalent maximum degree of cooling, or prevented warming, in the Polar Regions.

A similar finding re polar temperatures and ice extent is elaborated upon and explained in greater detail in [21], which examines the meridional heat flux as the mechanism for heat transfer from the Tropics towards the Polar Regions, and a major factor in the Arctic Ice melt. The ice-albedo feedback (e.g. [20]) has also been suggested as an increasingly important mechanism in the loss of Arctic ice and is discussed below.

Other studies [9–11] have also examined the possibility that MCB might be able to slow or even halt polar sea-ice loss. Rasch *et al*. [9] indicate that seeding 70% of maritime stratocumulus clouds could halt the loss of Arctic sea ice caused by a CO_2_-doubling (from 355 to 710 ppmv), and in the case of Antarctica increase the net sea-ice coverage. These GCM results indicate that MCB could be significant in this regard. However, they were based on producing enough cooling to maintain sea ice after compensating for warming from a doubling of CO_2_. This required cloud seeding over a significant (variable) fraction of the Earth's oceanic area, much of which is far away from the Polar Regions. Consideration is now being given to the possible efficacy of seeding a much smaller fraction of all marine clouds globally in more localized regions, either in the Tropics where the greatest natural cloud density occurs, or close to the Poles, to which ocean currents may carry cooler water most directly, to restore sea ice under current or near-term conditions.

In addition, it seems possible that methane release from the thawing of sub-sea permafrost, arising from ocean-summer warming due to sea-ice retreat, could at some stage produce a dangerous increase of atmospheric methane levels [22,23]. If so, a regional MCB seeding programme could in principle produce a localized surface cooling which might prove to be adequate to prevent further increase.

**Figure 4.**
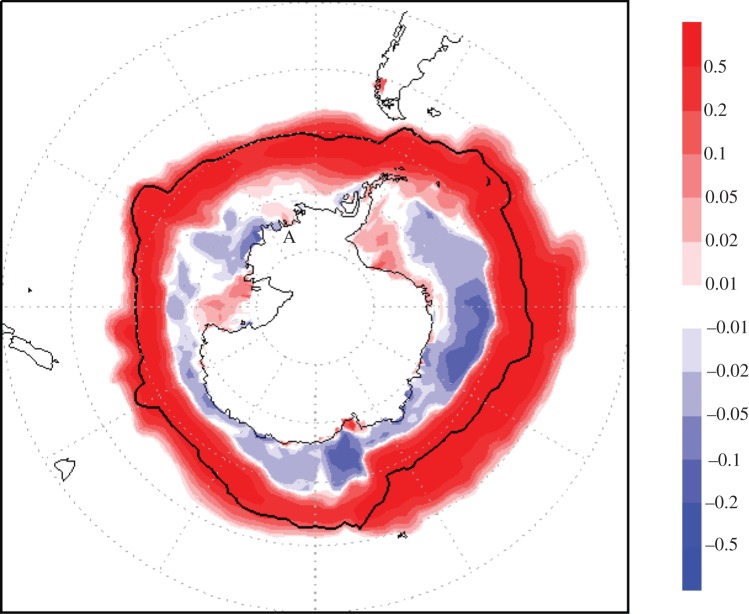
Marginal change in sea-ice cover for climate change (560 ppmv CO_2_) plus MCB All-Ocean seeding versus control (440 ppmv CO_2_). The black line represents the furthest extent North of the ice edge in the simulation.

In the Antarctic, MCB 3-region seeding does not show an appreciable effect. All-ocean seeding (MCBA) was also modelled for that region and is examined here. Figure 4 illustrates the net change in ice cover after seeding 100% of all possible ocean clouds at 560 ppmv CO_2_. This extends a similar, but less detailed, finding (fig. 2*c*,*e*, [9]) where significant increases in Antarctic ice cover were also found at seeding levels of both 40% and 70% of all clouds, at 710 ppmv CO_2_ versus, a control of 355 ppmv CO_2_.

In the South Polar Region shown in figure 4 (MCBA), the unobstructed Polar Vortex causes a significant increase in ice cover within a large annular ring of ocean, some reduction in a second ring closer to the continent including some areas of coastline, and local increases in ice cover in a number of coastal areas, several of which contain major ice shelves [24]. Mass flux studies show that two of the largest ice shelves, in the Ross and Weddell Seas, are actually increasing now and that the most significant loss of mass is currently occurring from glaciers, principally those draining into the Amundensen Sea [25]. The most important role of ice shelves with regard to sea level appears to be to stabilize inland glaciers.

Two recent studies of the Thwaites Glacier Basin predict that the collapse of the West Antarctic Ice Sheet is now inevitable [26,27]. The Thwaites Glacier is described as the lynchpin for the entire West Antarctic Ice Sheet. The narrow neck of the glacier is supported by a marine ice shelf, located in the Amundensen Sea.

Figure 4 shows the MCBA simulation, which produces a localized increase in sea ice in the Amundensen Sea, which corresponds with the location of the ice shelf at the base of the Thwaites Glacier (A in figure 4). This raises a general question as to whether the application of MCB could achieve a sustained temperature reduction and/or increase in ice cover in key areas within that region: and if so, whether a selective increase in localized sea-ice cover could be sufficient to delay or avert such a collapse, and the concomitant increases in sea level which have been predicted.

The localized phenomena illustrated in figure 4 may or may not itself actually indicate a viable approach to glacier preservation using MCB. However, the focus on one particular key ice shelf gives rise to another idea. The marine ice shelf supporting the Thwaites Glacier is reportedly being eroded from underneath by warmer ocean currents. If MCB proves to be effective, and if the ocean circulation patterns are stable, it may be possible to cool the ocean surface where the warmer currents originate, using MCB in an almost ‘surgical’ manner at local or regional scales.

The arguments just presented must be considered provisional and further research is required in order to determine whether MCB might be capable of producing a regionally directed cooling, either alone or in concert with other global measures capable of restoring stability to Antarctic ice sheets.

### (b) Hurricane weakening via marine cloud brightening

Tropical cyclones (also named typhoons, hurricanes and cyclonic storms in various basins) are major meteorological occurrences, but they are typically too small to be resolved in current climate models. However, climate change is accepted as a possible reason for an observed increase in the amount of power dissipated by North Atlantic hurricanes [28]. Much of this increase in power is attributed to an increase in SSTs in the equatorial regions [29–32], although some have argued that there may well be fewer storms, each of which dissipates a larger amount of energy [33]. However, more than 98% of tropical cyclones' formation occurs where the SSTs are over 25.5°C [34]. All these publications link an increase in intensity of tropical cyclones with an increase of SSTs caused by global warming. Our hypothesis is that MCB may prove capable of significantly lowering SSTs and hence reducing the energy available to tropical cyclones, so reducing their power or intensity [35]. The cooler waters generated by the cloud brightening are moved by existing currents around the globe and in particular into the hurricane development regions. An alternative or additional method of cooling surface waters could be the ingenious downwelling mechanism advanced by Stephen Salter (2011, personal communication).

We stress, however, that we recognize that the formation and development of hurricanes involves much more complexity than is afforded by consideration only of SSTs. A rigorous treatment of such development incorporates many other parameters. We do not regard our arguments and assumptions as quantitatively precise. Results [30,31] indicate that under present climatic conditions, an increase in SSTs does correlate well with tropical cyclone intensification. Future climate change projections indicate that although the Atlantic SSTs will warm rapidly, upper level tropospheric temperatures will also rise. Combined with increased vertical wind shear, these two factors are detrimental to further hurricane intensification (e.g. [36]) and the potential for future intensification is much more complicated than summarized here.

**Figure 5.**
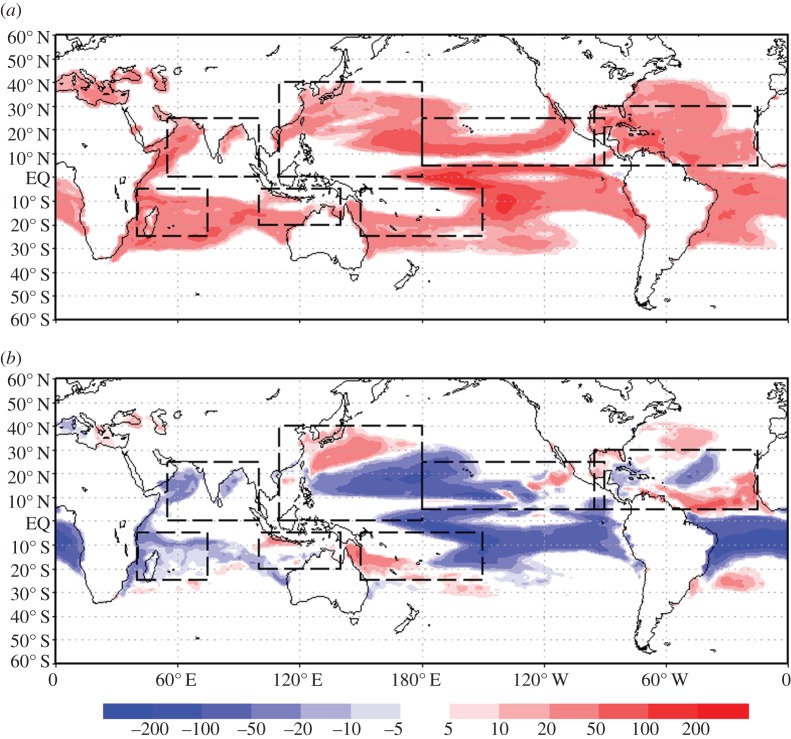
Difference in number of days per year where the temperature is above 26°C between: (*a*) [CC], (CO_2_=560 ppm) and CON, (CO_2_=440 ppm) simulations and (*b*) climate change plus MCB (three region seeding, CO_2_= 560 ppm) simulations. The simulated displayed time span is 2070–2090 and the boxes show seven tropical cyclone basins.

One approximate way of identifying a potential increase in the power of storms or duration is to count the number of days per year where the SST is above 26°C. The difference in this number between the CC and CON simulations is shown in figure 5. Seven basins were chosen, approximately encompassing the regions where the majority of these storms are generated. In all seven basins, there is an increase in the number of days above 26°C, which will result in more intense cyclones [33,34,36,37] and a larger global atmospheric meridional heat flux [21]. In the case of MCB and the control simulations, there is a notable reduction in the number of days above 26°C in three Indian Ocean basins. In the Eastern Pacific, the signal is mixed, with increases and decreases near the coast. This is probably an unphysical result and is likely to be caused by the poor resolution of the inter-tropical convergence zone. In the Western Pacific, the formation regions for tropical cyclones have fewer days above the 26°C limit. However, the coastal regions are more regularly warmer, which could lead to intensification just before landfall. In the Atlantic, African Easterly Waves are important in the formation of storms and they have more time to be become tropical cyclones storms, fully developing in warmer coastal waters of the Gulf of Mexico or Caribbean.

Thus, our basic idea regarding hurricane weakening, which as discussed above and in [33], is to use MCB seeding of marine stratocumulus clouds to cool ocean surface waters in regions where the genesis and early development of hurricanes occurs, or areas from which ocean currents flow to them. Our preliminary GCM modelling indicates that judicious seeding of maritime stratocumulus clouds, even in the three regions away from the hurricane development area, may reduce significantly the number of days in a year where the SSTs are above the 26°C limit. This point is illustrated in figure 5. In all seven basins, there is an increase in the number of days above 26°C, which will result in more frequent and intense cyclones [28–34] and a larger global atmospheric meridional heat flux [21].

The observationally based relationships between SST and maximum wind speed [37,38], together with our computed MCB-based SST-reduction values, allow us to make rough estimates of the associated hurricane category change. For parameter values used in our computations, a 1-category change is predicted. Much more work on this topic is required.

### (c) Reduction of coral bleaching via marine cloud brightening

Increases in thermal stress—typically calculated as degree heating weeks (DHWs)—have been accompanied by increases in coral bleaching events over the last few decades. This appears to be linked in large measure with rising SSTs [39–41]. Continued warming and associated increases in coral bleaching have been predicted throughout this century. Our GCM results [39] suggested that under the CC simulation (double pre-industrial CO_2_ conditions), the increases in coral bleaching in the three-reef regions studied (Caribbean, French Polynesia and Great Barrier Reef) were eliminated when MCB was applied. Here, we show an example of the localized spatial impacts of MCB in reducing the thermal stress that would occur between the control (CO_2_=440 ppm) and CC (CO_2_=560 ppm) simulations between years 2070 and 2090. Thermal stress is the accumulation of heat stress above some temperature threshold over a 12-week period [40]. It represents the time integral of any SST anomaly, which provides the source of the stress to the coral. We applied a threshold of n × SD_max_, where the empirical coefficient n, following [41], is taken as 2.45 and SD_max_ is the standard deviation of the annual maxima over the climatological period. The average thermal stress (DHW) was calculated for each of the 20-year model runs.

**Figure 6.**
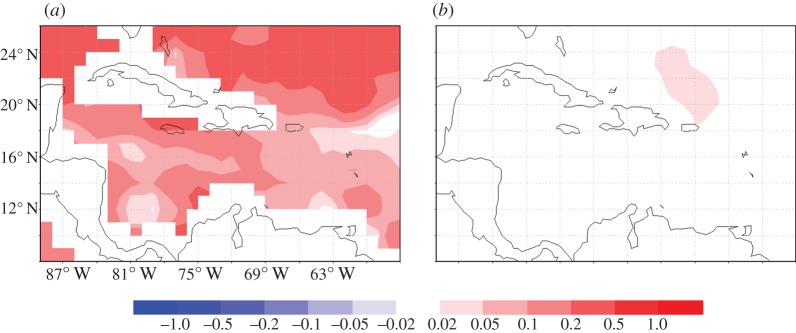
(*a*) Difference in the 20-year average DHW (2.45*s.d. method) between the Climate Change (CO_2_=560 ppm) and control (CO_2_=440 ppm) simulations between 2070 and 2090 for the Caribbean. (*b*) Difference in the 20-year average DHW (2.45*s.d. method) between the Climate Change and MCB (three region seeding, CO_2_=560 ppm) simulations between 2070 and 2090 for the Caribbean.

In all three regions, Caribbean, Great Barrier Reef and Polynesia, there is an increase in DHW in the Climate Change simulations. However, when MCB is applied, the DHWs are significantly reduced in each case. This conclusion, for the Caribbean, is illustrated in figure 6.

## 3. Discussion: other issues related to marine cloud brightening and regionality

Most discussion of MCB to date has focused on producing a negative forcing of roughly 4 W m^−2^ necessary to counteract the effects of a doubling of CO_2_. The current additional net forcing above a pre-industrial baseline is estimated at roughly 1.5 W m^−2^. The portion of that forcing which may be responsible for the effects that are now beginning to be observed in the Arctic, Antarctic and other regions might be of the order of 1 W m^−2^. If new CO_2_ emissions could be curtailed more rapidly than has been anticipated, and the CO_2_ level could be rapidly stabilized at the low end of the possible scenarios, we would still face effects of warming which are already being described as inevitable and catastrophic. It might be possible to use MCB to address or even rectify such regional issues.

The amount of seeding which would be required to produce a much smaller amount of negative forcing is nonlinear, such that producing 25% as much cooling might only require 5–15% as much seeding [6]. At vastly reduced levels of seeding, it is also likely that any adverse consequences—such as the perturbation of rainfall over terrestrial regions—which have been seen when modelling a doubling of CO_2_, might also be vastly reduced or eliminated. Given the serious consequences, which are already beginning to occur from observed warming, reduced seeding scenarios deserve further study.

It may be useful to make some distinctions between the location of seeding, and the location of the resulting reduction in regional SSTs, for various possible regional implementations of MCB. For example, a localized seeding to reduce SSTs in proximity to a coral reef might represent one end of a spectrum, while regional seeding in the vicinity of a cyclone basin to reduce SSTs within that particular basin might represent a larger regional application. At the opposite end of the spectrum, the examination of the Arctic has so far actually focused on the effects of meridional heat transfer and the zonal amplification of global temperature effects, which appear to disproportionately reduce temperatures in the Polar Regions as a result of seeding in warmer geographically distant regions. One MCB seeding pattern, which has been modelled in relation to the Arctic, is described as ‘three region seeding’, because there are three distinct regions of ocean where seeding is expected to be most effective, but those three seeding regions are geographically widely separated and far from the Arctic. By contrast, there may, hypothetically, be a secondary approach to reducing SSTs in the Arctic, which might be more similar to localized applications. Ocean clouds in various regions that are in closer proximity to the Arctic and located upstream on prevailing currents could theoretically be seeded to reduce SSTs in the Arctic. In the Southern Hemisphere, if a highly directed seeding aimed at selectively cooling ocean waters flowing towards a particular ice shelf were to be effective, such a seeding regimen might have more in common with the localized example of a coral reef, than with a three region seeding pattern aimed at addressing Arctic sea ice.

Although we have focused herein—and also in previous MCB papers—on the production of a cooling on planetary and regional scales, it is clear that the seeding of marine stratocumulus could theoretically be modified to produce fewer, larger drops, which would be expected to produce a warming, instead. This would have possible value only on regional scales. This idea has two possibly useful characteristics. (i) The advertent production of a localized warming could perhaps be used to enhance rainfall in regions where it is particularly needed. (ii) The capacity to produce a warming as well as a cooling adds to the flexibility of our cloud seeding technique, and perhaps could be used to fine-tune the flows of heat, air and water. The capacity of MCB to produce—in specified circumstances—a warming rather than a cooling, has been discussed by Alterskjær *et al*. [14] and—in much less detail—by Latham [4,8].
